# Case Report: “Gourd-Shaped” Heart Strangled by Localized Annular Calcification of the Left Ventricle: A Rare Case of Constrictive Pericarditis

**DOI:** 10.3389/fcvm.2022.834262

**Published:** 2022-02-04

**Authors:** Zhiyun Yang, Hui Wang, Qiang Lv, Xin Du, Junming Zhu, Jianzeng Dong

**Affiliations:** ^1^Department of Cardiology, Beijing Anzhen Hospital, Capital Medical University, Beijing, China; ^2^National Clinical Research Center for Cardiovascular Diseases, Beijing, China; ^3^Department of Radiology, Beijing Anzhen Hospital, Capital Medical University, Beijing, China; ^4^Department of Cardiac Surgery, Beijing Anzhen Hospital, Capital Medical University, Beijing, China; ^5^Department of Cardiology, The First Affiliated Hospital, Zhengzhou University, Zhengzhou, China

**Keywords:** multimodality imaging, heart failure, cardiac magnetic resonance feature tracking, pericardial calcification, constrictive pericarditis

## Abstract

We report a rare case of a 43-year-old woman with calcific annular constrictive pericarditis (CP) encircling the basal segment of the right ventricle and the mid-segment of the left ventricle (LV) lateral wall. Over time, localized calcification has caused LV to be tightly strangled and shaped like a gourd. However, multimodality imaging confirmed no significantly clinical constriction associated with decreased cardiac movement and function. Additionally, cardiac magnetic resonance feature tracking confirmed the relatively preserved diastolic function and the characteristic “plateau” pattern of CP. The treatment strategy of this case is challenging and dialectical.

## Introduction

Constrictive pericarditis (CP), caused by primary (idiopathic, posterior pericarditis or linked to rheumatism) or secondary factors (after heart surgery or radiation therapy), is characterized by pericardial adhesions, thickening, or calcification. The inelastic pericardium inhibits cardiac filling, causing cardiac diastolic filling dysfunction and diastolic heart failure (1). CP diagnosis may be challenging and requires multimodality imaging assessment: echocardiography, computed tomography (CT), and cardiac magnetic resonance (CMR) for structural and movement evaluation; catheterization to evaluate hemodynamics; myocardial strain rate analysis to assess myocardial deformation and function, etc. (2, 3). For patients with thickened and calcified pericardium, pericardiectomy is a potentially curable intervention that can improve prognosis. Most pericardial calcifications removed by pericardiectomy were found on the inferior and diaphragmatic surface, anterior right ventricular surface area, and atrioventricular groove posteriorly and over the infundibulum anteriorly (4, 5). While a few cases of pericardial calcification in localized areas have not undergone surgery, lesions of fibrotic or calcified pericardium have been retained *in situ* (6–8). Here, we present a rare case of a middle-aged woman who had calcified annular constrictive pericarditis with localized strangulation in the mid-segment of the left ventricle (LV) lateral wall and a “gourd-shaped” heart.

## Case Report

**Figure 1:**
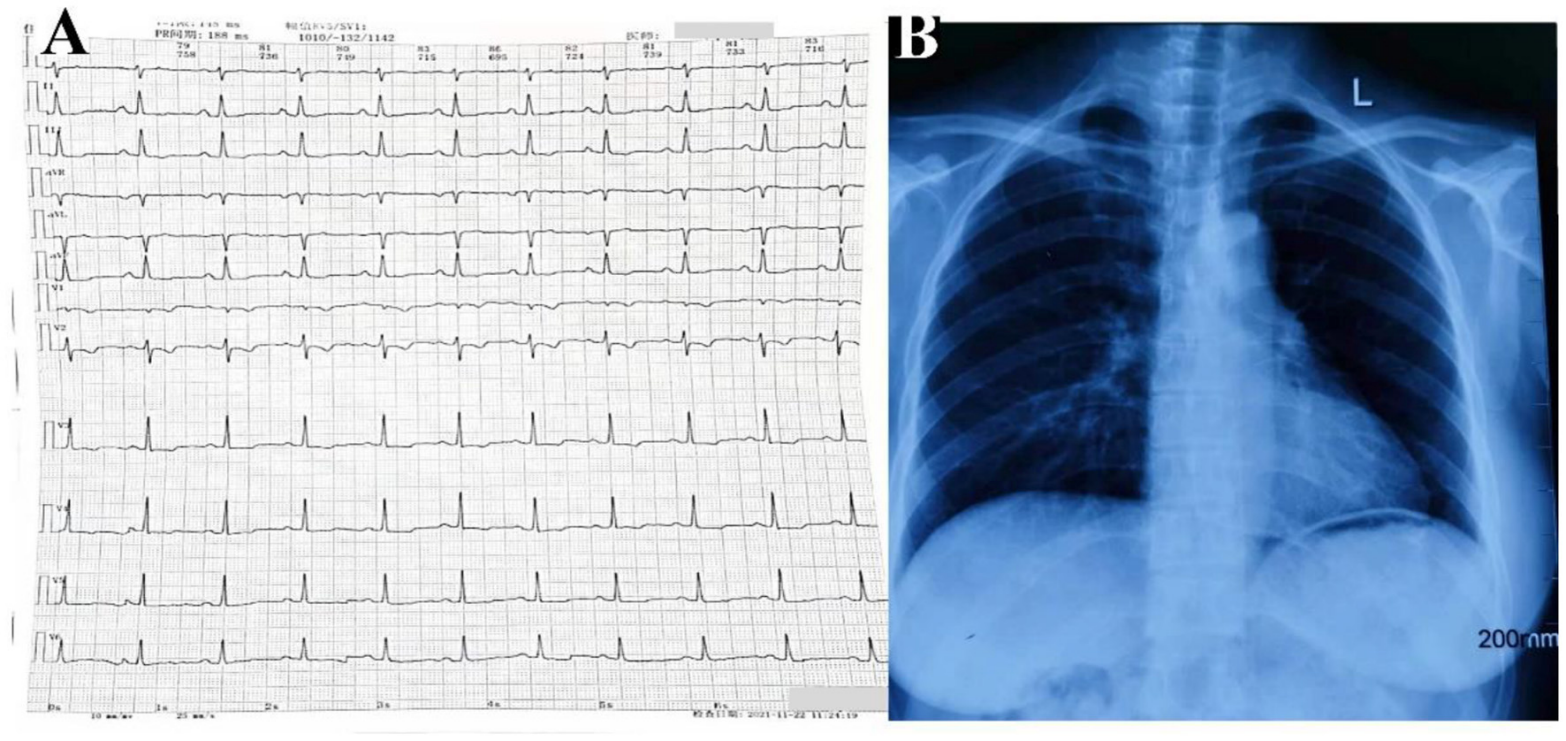
Electrocardiogram **(A)** and chest radiogram **(B)** of calcific annular constrictive pericarditis. Electrocardiogram **(A)** showed sinus rhythm and secondary ST-segment and T-wave changes caused by pericardial calcification, and chest radiogram **(B)** demonstrated pericardial calcification was not observed obviously.

**Figure 2:**
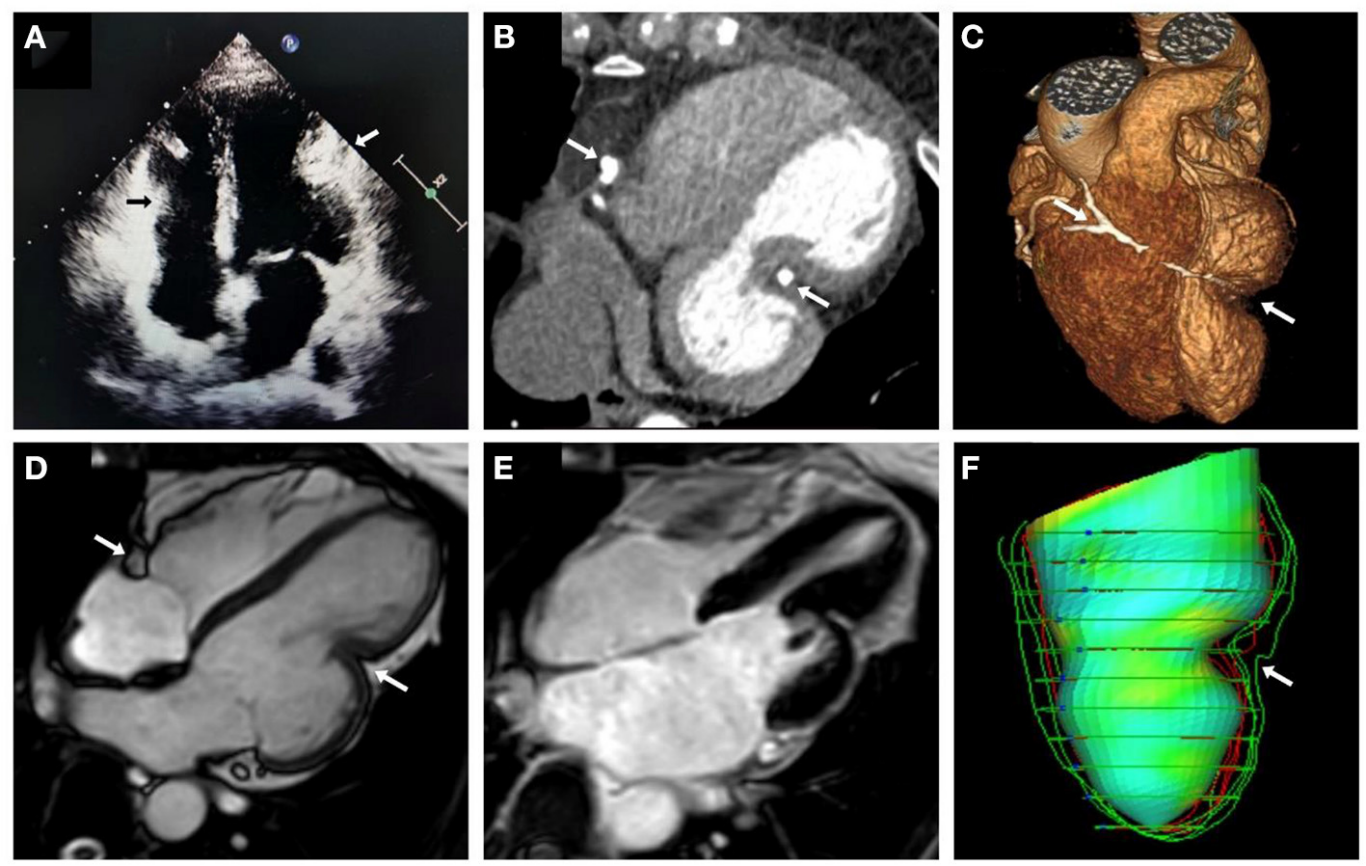
Multimodality imaging of calcific annular constrictive pericarditis. Echocardiographic in apical 4-chamber view **(A)**, CT in 4-chambers view **(B)**, 3D volume rendering view **(C)**, and CMR cine imaging in 4-chambers view **(D)** revealed calcific annular constrictive pericarditis trapping in the right atrioventricular groove and the mid-segment of LV lateral wall (arrows) and the altered shape of “gourd-shaped” heart. CMR delayed enhanced imaging **(E)** showed no obvious abnormal findings. LV feature tracking model **(F)** also showed localized strangulation in LV and abnormal heart shape changes. CMR, cardiac magnetic resonance; CT, computed tomography; LV, left ventricle.

**Figure 3:**
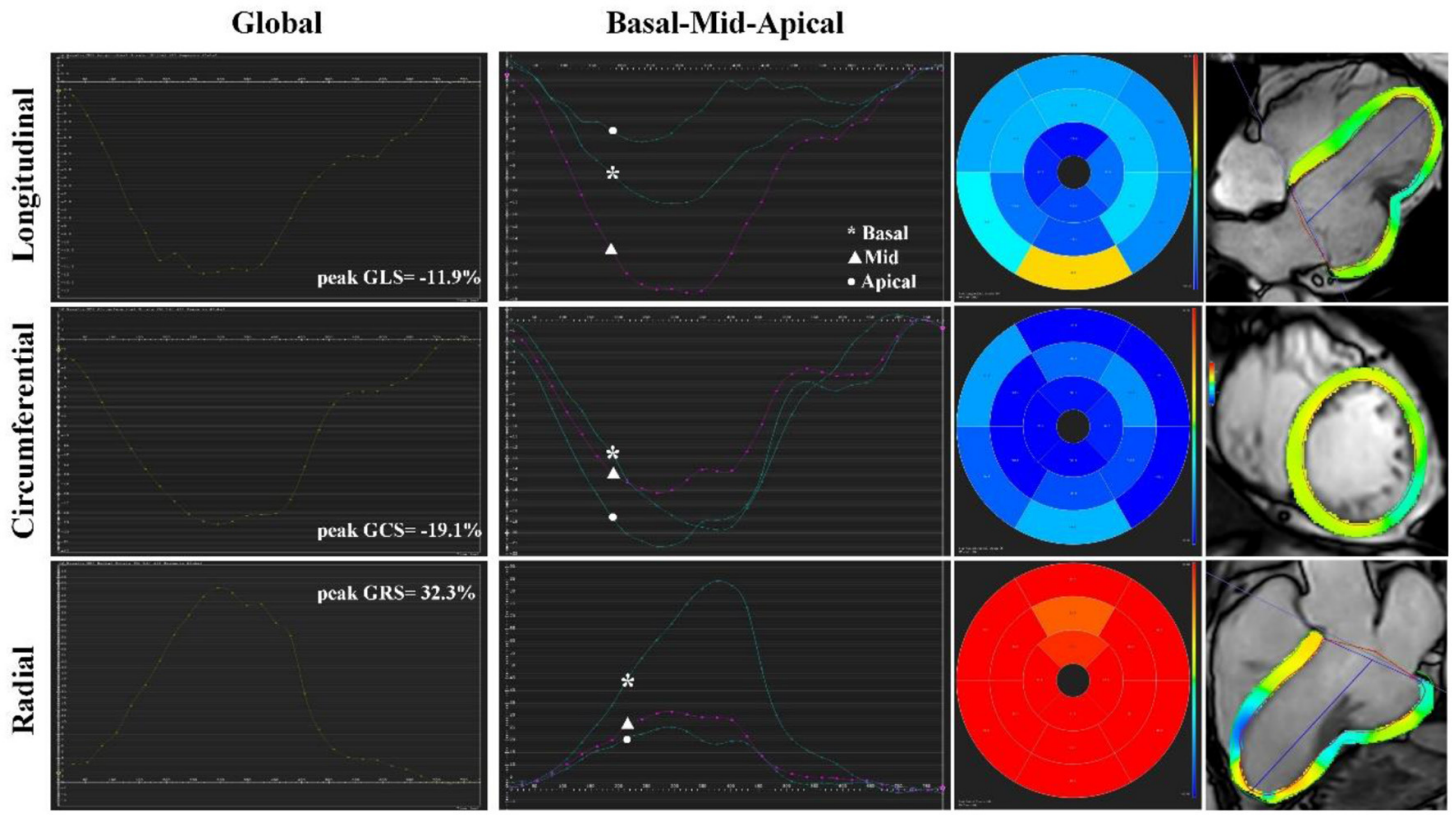
Cardiac magnetic resonance feature tracking strain rate analysis of calcific annular constrictive pericarditis. CMR-FT revealed LV global myocardial strain rate, LV global and regional basal-mid-apical segment time-strain curves, and bull's-eye plots in longitudinal, circumferential, and radial direction. The characteristic “plateau” pattern of CP could be observed in LV global and regional mid-segment time-strain curves. CMR-FT, cardiac magnetic resonance feature tracking; CP, constrictive pericarditis; GCS, global circumferential strain; GLS, global longitudinal strain; GRS, global radial strain; LV, left ventricle.

A 43-year-old woman was referred to Anzhen Hospital (Capital Medical University of Beijing, China) for a definite diagnosis and diastolic function evaluation. With a 7-year history of discovering pericardial calcification, she had no obvious symptoms or signs of systemic congestion and heart failure, only intermittent mild edema of both lower limbs. Laboratory examination showed that she had normal inflammatory marker levels, liver and renal function levels and plasma B-type natriuretic peptide level, and laboratory examination also excluded tuberculosis and rheumatism. Electrocardiogram showed sinus rhythm and secondary ST-segment and T-wave changes caused by pericardial calcification, and chest radiogram demonstrated pericardial calcification was not observed obviously (Figure 1). Transthoracic echocardiography revealed localized cardiac constriction for the right atrioventricular groove and LV lateral wall due to nodular masses (Figure 2A). Additionally, echocardiography indicated that normal size and systolic function of LV; bi-atrial enlargement; paradoxical septal motion with ventricular septal shift during respiration; early to late diastolic transmitral flow velocity (E/A) >1; mitral medial annulus e' velocity >8 cm/s; and mitral lateral annulus e' velocity (14 cm/s)<mitral medial annulus e' velocity (17 cm/s). Pulsed-wave Doppler showed respiratory variation in transmitral flow and the inferior vena cava was dilated (3 cm) without inspiratory collapse. All the above findings were consistent with the diagnosis of pericardial constriction. CT images clearly indicated calcified annular constrictive pericarditis encircling the basal segment of the right ventricle (RV) and the mid-segment of LV. As a result, the heart was tightly strangled and shaped like a gourd in CT imaging (Figure 2B) and 3D volume rendering view (Figure 2C). CMR revealed localized strangulation at the mid-segment of LV lateral wall, consistent with echocardiography and computed tomography findings. There was no significant reduction in the movement of each LV segment in cine imaging; no obvious abnormal findings in delayed enhanced imaging; and LVEF remained at 58% in CMR (Figures 2D,E).

To provide a more accurate assessment of myocardial deformation and LV diastolic function, we performed cardiac magnetic resonance feature tracking (CMR-FT) strain rate analysis for this patient. We analyzed LV global myocardial strain rate and LV global and regional basal-mid-apical segment time-strain curves, and obtained a three-dimensional LV feature tracking model. CMR-FT analysis indicated that the peak global longitudinal strain (GLS) was −11.9%, the peak global circumferential strain (GCS) was −19.1%, and the peak global radial strain (GRS) was 32.3%. Additionally, the characteristic “plateau” pattern of CP could be observed in LV global and regional mid-segment time-strain curves (Figure 3). In addition, there was localized strangulation in the mid-segment of LV lateral wall and a “gourd-shaped” heart in LV feature tracking model (Figure 2F). Hemodynamic evaluation elucidated and corroborated the effect of constrictive pericarditis on left ventricular filling. Multimodality imaging confirmed that the patient was definitely diagnosed with calcified annular constrictive pericarditis caused by constriction due to ring-shaped and localized calcified pericardium. Due to the location and shape of the calcification, relatively preserved diastolic function, and patient's desires, pericardiectomy has not yet been performed, and diuretic therapy (temporary oral torasemide 20 mg) has been prudently permitted when lower limbs edema occurs. Regular visits will follow up with this patient, and surgical treatment will be performed if necessary.

## Discussion

The uniqueness of this case is that calcified annular constrictive pericarditis is an extremely rare form of localized constrictive pericarditis. More precisely, the calcification, in this case, surrounds the right atrioventricular groove and the mid-segment of the LV lateral wall, rather than typically encircling the bilateral atrioventricular groove as reported by Carabelli et al. (9). Gradually, localized pericardial calcification, in this case, has resulted in an obvious stranglehold and altered shape of LV, but did not cause significant clinical constriction with decreased cardiac movement and function. For most patients with chronic and progressive CP, the radical treatment is surgical pericardiectomy. In ESC Guidelines, pericardiectomy is recommended for highly symptomatic patients of CP [New York Heart Association (NYHA) functional class III-IV] (recommendation I-C) and should be cautiously considered in patients of CP with mild symptoms or those with an advanced stage of the disease (cachexia, atrial fibrillation, low cardiac output, hypoalbuminemia, liver dysfunction), those showing myocardial dysfunction or significant kidney failure, and those with a disease secondary to radiotherapy (10). Pericardiectomy is a relatively safe procedure and a potentially curative treatment with an average perioperative mortality rate of 6% (11) and a reported 5–7 years survival rate over 80% (12). The best surgical procedure for pericardiectomy is median sternotomy which allows for easy dissection of diaphragmatic, posterior pericardium to the left phrenic nerve, vena cava, right atrium, and ventricle (13). Although anterior pericardiectomy makes dissecting LV easier than median sternotomy, it is not recommended yet (14). Unfortunately, this patient's LV calcification is as narrow as a ring, tightly attached to LV lateral wall and straddling the coronary arteries. The above-mentioned factors may increase the difficulty and risk of the operation, such as difficulty in peeling off pericardial calcification, bleeding, coronary artery damage, and myocardial atrophy following prolonged constriction.

On the other hand, multimodality imaging evaluation of this patient confirmed that LVEF and myocardial movement were not significantly reduced, and no evidence of necrosis or fibrosis was observed in the myocardium. Through CMR-FT strain rate analysis, we found that LV global strain rate of the patient demonstrated the relatively preserved diastolic function (the patient vs. age- and sex-matched healthy volunteers: peak GLS: −11.9 vs. −15.2 ± 3.7%; peak GCS: −19.1 vs. −23.4 ± 2.27%; peak GRS: 32.3 vs. 43.5 ± 9.8%). For most cases of CP, during the process of diastolic filling, LV stiffness and pericardial restraint will lead to increased LV afterload and impaired motor function, which ultimately leads to a significant reduction in circumferential deformation (presented as GCS, reflects subepicardial myofibres). However, due to the relatively preserved myocardial motion and function, this patient did not have a significant decrease in global strain rate. In addition, the characteristic “plateau” pattern of CP in LV global and regional mid-segment time-strain curves corresponded to the location of calcified pericardium (15). Balancing risks and benefits of this patient, we decided to postpone the operation, follow up the patient regularly, and pay close attention to the progressive course.

## Conclusions

Our case highlights the critical role of multimodality imaging in CP definite diagnosis, observation of the location and shape of pericardial calcification, and evaluation of myocardial movement and function. In addition, for chronic CP patients with a rare location of calcification and relatively preserved diastolic function, the timing and postoperative prognosis of pericardiectomy are unclear, which requires additional research to confirm.

## Data Availability Statement

The original contributions presented in the study are included in the article/supplementary material, further inquiries can be directed to the corresponding author/s.

## Ethics Statement

The studies involving human participants were reviewed and approved by the Institutional Ethics Committee of Anzhen Hospital, Capital Medical University of Beijing, China (Approval No. 2006003x). The patients/participants provided their written informed consent to participate in this study.

## Author Contributions

JD was the guarantors of the integrity of the entire study. HW and QL are responsible for the study concepts and design. ZY prepared the first draft of the manuscript, which was critically revised by XD, JZ, and JD. All authors contributed to the article and approved the submitted version.

## Funding

This work was supported by the National Natural Science Foundation of China (Grant No. 82170381).

## Conflict of Interest

The authors declare that the research was conducted in the absence of any commercial or financial relationships that could be construed as a potential conflict of interest.

## Publisher's Note

All claims expressed in this article are solely those of the authors and do not necessarily represent those of their affiliated organizations, or those of the publisher, the editors and the reviewers. Any product that may be evaluated in this article, or claim that may be made by its manufacturer, is not guaranteed or endorsed by the publisher.
